# Factors Influencing Depression and Anxiety among Black Sexual Minority Men

**DOI:** 10.1155/2011/587984

**Published:** 2011-09-18

**Authors:** Louis F. Graham, Robert E. Aronson, Tracy Nichols, Charles F. Stephens, Scott D. Rhodes

**Affiliations:** ^1^School of Public Health, University of Michigan, Ann Arbor, MI 48109-2029, USA; ^2^Department of Public Health Education, University of North Carolina Greensboro, Greensboro, NC 27402, USA; ^3^Department of Education and Volunteer Services, AID Atlanta, Atlanta, GA 30309, USA; ^4^Department of Social Sciences & Health Policy, Wake Forest University Health Sciences, Winston-Salem, NC 27157, USA

## Abstract

The primary aim of this study was to examine the relationships between depression and anxiety, and ethnic and sexual identity development, and discrimination and harassment (DH) among Black sexual minority men. Additional aims were to determine whether an interaction effect existed between ethnic and sexual identity and whether coping skills level moderated these relationships. Using an observational cross-sectional design, 54 participants recruited through snowball sampling completed self-administered online surveys. Stepwise multiple regression analysis was used. Sixty-four percent of the variance in depression scores and 53% of the variance in anxiety scores were explained by DH and internalized homonegativity together. Thirty percent of the sample had scale scores indicating likelihood of depression and anxiety. Experience of DH and internalized homonegativity explained a large portion of the variability in depression and anxiety among Black sexual minority men. The study showed high prevalence of mental distress among this sample.

## 1. Introduction

Research and theoretical suppositions suggest that Black sexual minority men (BSMM) may experience more depressive symptoms and anxiety than their male heterosexual and Black female counterparts and at minimum parallel those of their white sexual minority counterparts. Little is known about factors that influence the psychosocial health of BSMM. The limited research conducted with BSMM, predominantly White samples of sexual minority men, and Black men with unspecified sexuality, however, indicates that unique concerns related to identity and exposure to violence and discrimination may play important roles. Also critical may be individual's internal and external resources that can serve as coping tools. The primary aim of this study was to examine the relationships between depression and anxiety, and ethnic and sexual identity development (operationalized as Black identity achievement and internalized homonegativity), discrimination and harassment (DH), and coping skills among BSMM.

Mental health disorders affect a considerable proportion of the general population in the United States [1] with roughly 21% and 29% of adults meeting Diagnostic and Statistical Manual of Mental Disorders, Fourth Edition criteria for a mood or anxiety disorder, respectively, over their lifetime [2]. General population investigations using random sampling have shown that homo-/bisexual men are more likely than heterosexual men to have mood and anxiety disorders [3–7]. In a meta-analysis, Meyer [8] concluded that the odds of gay and bisexual men experiencing an anxiety or mood disorder over their lifetime were twice those of straight men. In a convenience sample, Cochran and Mays [9] found a 32.6% prevalence of depression among BSMM. Also, in other studies African American gay men were observed to have an increased probability of feeling anxiety and isolation but were unlikely to seek professional help [10, 11].

Studies show longstanding trends in the high rates of violence, discrimination, and harassment (VDH) perpetuated against sexual minorities [12]. Graham and colleagues' [13] study found a negative effect of violence and discrimination on the mental health status of BSMM. Similarly, Crawford et al. [14] found negative associations between experiences of perceived racist events and life satisfaction and anxiety among BSMM. Finally, in a probability sample of 912 Latino men who had sex with men (MSM), recruited from social venues in New York, Miami, and Los Angeles, 10% reported that they had experienced violence as an adult because of their sexual orientation or femininity [15]. Other factors influencing mental health outcomes among BSMM may include racial identity development, internalized homonegativity, and coping strategies.

Racial and sexual identity components are core for BSMM [16, 17]. Graham et al. [13] explored the psychosocial health of BSMM and found that BSMM were challenged in developing a healthy identity. They concluded that struggles related to the unique experience of being BSMM such as negative attitudes and beliefs concerning their race and sexuality as well as gender conformity pressure contributed to depression and anxiety. A study by Crawford et al. [14] of African American gay and bisexual men found that BSMM with a positive racial identity were significantly less likely to have mental distress and more likely to have greater life satisfaction and self-esteem than BSMM with less positive racial identity.

Positive racial identity is denoted as “the process of development by which individual members of various socioracial groups overcome the version of internalized racism that typifies their group in order to achieve a self-affirming and realistic racial-group or collective identity” [18]. According to Helms' [19] model of Black racial identity, the least affirming status is preencounter. The next racial identity phase, encounter, commences once the individual has a personal thought-provoking experience with race that leads the individual to question his racial identity. The third phase, immersion, involves coming to understand the worth and significance of the individual's ethnic heritage. The last stage, internalization, is characterized by affirming and valued perception of oneself. Pierre and Mahalik [20] examined Black racial identity development as a predictor of psychological distress and self-esteem among a college and community sample of 130 Black men of undocumented sexuality aged 18 to 25 years. They found that preencounter and immersion racial identity attitudes were associated with more psychological distress and less self-esteem, while internalization attitudes were associated with greater psychological well-being.

Theoretically, internalized homonegativity hinders the course of healthy identity development [21]. Internalized homonegativity can be described as negative, disapproving, or repudiating views or perceptions of homosexuality or related sexuality components, that persons with a same sex orientation have accepted, believed, or taken on from an external source [22, 23]. Rosser et al. [24] examined the relationship between homosexuality, internalized homonegativity, and mental health in MSM and found that internalized homonegativity, but not homosexuality, was an important predictor of depression in homosexual men.

Coping has been defined as the views and behaviors individuals use to deal with burdens they identify as exceeding their resources [25]. Coping mechanisms include efforts to change the pathway stress takes (problem-focused coping) and efforts to control emotional reactions to stressors (emotion-focused coping). A study by Peterson and colleagues [26] found that psychosocial resources, including optimum social support and spirituality, mediated the effects of stressors on depressive mood among Black MSM. An investigation that included both Black and White homosexual men found that BSMM were more likely to use disengaged coping than White gay men [27]. Other investigations have found a statistical relationship between perceived racism and disengaged coping [28, 29]. Finally, a study of a diverse sample of gay men by David and Knight [30], found that though BSMM were more likely to use disengaged coping styles, they did not appear to experience more negative mental health outcomes as a result.

Few studies have examined the relationships of DH, racial and sexual identity development, and coping with depressive symptoms and anxiety among BSMM. This study therefore examined these relationships. This study focused on the following research questions.

1. Are internalized homonegativity and DH significantly positively associated with depression and anxiety?
2. Is Black identity achievement significantly negatively associated with depression and anxiety?
3. Is there an interaction effect between internalized homonegativity and Black identity achievement on depression and anxiety?
4. Does coping skill level moderate the associations between depression and anxiety, and internalized homonegativity, DH, and Black identity achievement?

Better understanding mental health determinants among BSMM will enable population health practitioners, medical service providers, and policymakers to help prevent mental distress and disorders, effectively promote overall psychosocial health, and intervene early in distress sequelae.

## 2. Methods

### 2.1. Design and Sampling

An observational cross-sectional study design was used. The University of North Carolina, Greensboro, served as the primary study site; however, participants were recruited and surveyed at a variety of sites. Volunteer snowball sampling was employed. The study was approved by the university Institutional Review Board (IRB). Posters, flyers, and palm cards were distributed at community facilities and events, and letters were drafted and sent to e-mail and physical address listservs.

Additionally, advertisements were placed in local newspapers and on social networking websites. The materials included a short description of the study, including its purpose and the contact information of study personnel, as well as a web link for the project. Men 18 years old or over who self-identified as being of African descent (Black, African American, etc.) and as men who had sex with, desired to have sex with, or eroticized sex with men were enrolled in the study. Informed consent was obtained from participants, and they were provided a unique identifier and password to complete a battery of surveys at a secured website accessed from their own personal computer or laptop computer provided for them. Participants were required to answer each question before moving to the next; the battery of surveys took between 30 and 45 minutes to complete, after which participants received a $25-gift card to Target.

### 2.2. Measures

#### 2.2.1. The Center for Epidemiologic Studies Depression Scale (CES-D)

The center for epidemiologic studies depression scale (CES-D) [31] was developed for use with general adult populations (aged 18 or older). This 20-item self-report scale measures depressive symptoms during the previous 7 days, and responses (0-rarely or none of the time, 3-most or all of the time) are summed. Sample items include “I felt sad,” “I felt lonely,” and “I felt fearful.” Positive items are reverse-scored, and the possible range of scores is zero to 60, with higher scores indicating more symptoms.

Cronbach's alpha for the CES-D in this study was .95. The CES-D may underestimate likelihood of depression given recent evidence indicating that the CES-D may not be an accurate screening tool for ethnic minorities [32]. Depression scores in different racial/ethnic subpopulations might be biased by response patterns that vary between racial groups, not because a community has more or fewer symptoms or disorders, but because the subpopulation articulates psychopathology in a manner not captured by measures normed primarily in a different ethnic group [33]. No better scale has been identified, and given its widespread use among Black and MSM populations, the CES-D was used in this study.

#### 2.2.2. State-Trait Anxiety Inventory (STAI-S)

The STAI-S [34], which was used to measure anxiety, is a 20-item questionnaire intended to evaluate current anxiety and has been used with African American populations. Sample items include “I feel calm,” “I feel tense,” and “I feel upset.” The STAI has two factors, anxiety-present and anxiety absent. Anxiety absent items are reverse-scored. Each item is rated from 1 (not at all) to 4 (very much so) to reflect the level of each affect statement, and responses are summed. 

Higher scores represent greater anxiety. The STAI-S has demonstrated satisfactory internal consistency and test-retest reliability in numerous studies [35]. Additionally, it has demonstrated satisfactory convergent and discriminate validity with other measures [36, 37]. Cronbach's alpha in this study was .96.

#### 2.2.3. Demographic Information Sheet

Demographic information sheet asked 10 questions focused on three components of sexuality (orientation, identity, and role), socioeconomic status (educational attainment and annual income), religious affiliation, age, and history of depression diagnosis.

#### 2.2.4. The Black Racial Identity Attitudes Scale (RIAS-B)

The black racial identity attitudes scale (RIAS-B) was developed to identify stage placement in the Cross model of minority identity development [38]. The model posits that, as African Americans become aware that they are oppressed, their attitudes toward themselves, their own group, other ethnic minority groups, and members of majority cultures take shape in a way that leads to a central sense of self [39].The tool consists of 50 statements to which participants are asked to respond using a Likert-type scale (1-strongly disagree, 5-strongly agree). Some statements indicate concrete actions, some are descriptive terms, and others are statements of personal values and beliefs. Items reference the current state. 

Subscales are scored by averaging items so that the respondent receives a scale score for each of four types of racial identity attitudes (preencounter, encounter, immersion/emersion, and internalization/commitment). The highest mean subscale score reflects placement at that particular racial identity attitude stage. Sample items include “the people I respect most are White,” “being Black just feels natural to me,” “and White people can't be trusted.” Cronbach's alpha for subscales have ranged from .51 to .80. In this study, Cronbach's alphas were Stage 1—*α* = .89, Stage 2—*α* = .56, Stage 3—*α* = .70, Stage 4—*α* = .86.

#### 2.2.5. Internalized Homonegativity Inventory (IHNI)

A 17-item revised version of the IHNI was used in this study. The IHNI was adapted for use among BSMM because it was originally validated on 241 gay men primarily of European descent [40], it has only been used once among a predominantly Black sample where potential validity issues were raised, and there were men in the current study who identified in diverse ways not referenced in the original IHNI (e.g., bisexual, SGL). Study investigators and a panel of experts assessed translation validity by examining face and content validity of items in the 3 subscales (personal homonegativity, gay affirmation, and morality of homosexuality). Language referencing homosexual and gay was broadened to encompass a wider range of behavior and identity, and two items were added to the personal homonegativity subscale: “I feel ashamed when I see or am around other sexual minority men who are obviously homo/bisexual or who are acting gay/SGL” and “I believe homo/bisexual men are weak.” 

Principal axis factor analysis with varimax rotation suggested deletion of 8 items (four from the first subscale, three from the second, and one from the third). The original tool and the modified tool were highly correlated with an *r* = .96, which establishes convergent validity in that the tools theoretically should be related to each other. Factor analysis revealed a slightly better performance of the altered IHNI as compared to the original in this sample. The revised IHNI factor solution resulted in a decrease of items that crossloaded and appeared slightly more meaningful in that it better reflected the hypothetical factor structure presented in the construct definition of internalized homonegativity.

Sample items include “I am proud to be homo-/bisexual,” “I believe that it is morally wrong for men to have sex with other men,” and “I sometimes resent my sexual orientation.” IHNI items reference current state, responses (1-strongly disagree to 6-strongly agree) are summed, and positive items are reverse-scored. Higher scores on the personal homonegativity and morality of homosexuality subscale and lower scores on the gay affirmation subscale represent more IHNI, where higher scores on the total IHNI represent greater internalized homonegativity. Cronbach's alphas for the adapted 17 item IHNI used in this study were total—*α* = .97, Factor 1—*α* = .95, Factor 2—*α* = .91, Factor 3—*α* = .89.

#### 2.2.6. Perceived Ethnic Discrimination Questionnaire-Community Version (PEDQ-CV)

The PEDQ-CV is used to evaluate perceived ethnic discrimination and was used in this study as a measure of discrimination and harassment (DH). This scale is a modification of the PEDQ-Revised B, developed by Contrada and colleagues [41] to evaluate perceived exposure to discrimination. To develop the community version, the original items were phrased in simpler language and adapted to reflect the everyday experiences of community-dwelling adults [42]. The lifetime discrimination scale (34 items) which includes four subscales (exclusion/rejection, stigmatization, discrimination at work/school, and threat/aggression) and the discrimination in different settings component of the PEDQ-CV were used. Sample items include “people ignored you,” “people do not trust you,” and “people actually hurt you.” 

Participants were also asked to indicate whether race, sexuality, both race and sexuality together, or one or the other, but they could not tell which, was primarily involved in their experience of each type of DH in the discrimination scale and in each community sector in the settings component. The measures are used to assess past-year experiences in social and interpersonal contexts. They have been used with Latino and Black subjects. The past-year discrimination responses (0-never happened to 4-happened daily) are summed, with higher scores representing more DH. Cronbach's alpha in this study was .98.

#### 2.2.7. Brief COPE

The brief COPE is a shortened adaptation of the COPE Inventory [43]. Comprised of 28 items that measure both active and disengaged coping styles, it includes 14 subscales (of 2 items each) that reflect coping activities. Sample items include “I criticize myself,” “I make jokes,” and “I learn to live with it.” Subscales were combined into two factors: disengaged coping and active coping, consisting of six and eight subscales, respectively; disengaged coping items were reverse-coded. 

Active coping includes use of emotional support, use of instrumental support, positive reframing, planning, humor, acceptance, and religion. Disengaged coping includes self-distraction, denial, substance use, behavioral disengagement, venting, and self-blame. Items ask respondents to consider how they usually feel, think, and respond given stressful or depressing situations or events; response options range from 0 (I usually do not do this at all) to 3 (I usually do this a lot). Cronbach's alpha in this study was .88.

## 3. Results

### 3.1. Demographics

Seventy-seven percent of participants indicated that they had sex with or desire to have sex with males only, 77% self-identified as gay, and 13% identified culturally as same gender loving (Table 1). Thirty-nine percent had completed a two-year degree or had some college education, and 31% had completed a four-year degree. The average annual income of participants was $25,275, with a range of $0 to $68,000. The average age was 31 years, with the youngest participant being 19 and the oldest 50; 50% designated their religious affiliation as Christian, and 33% designated themselves as spiritual. Thirty-three percent of participants said they had been diagnosed with depression by a healthcare professional, 30% had CES-D scores above 15 indicating likelihood of depression, and 33% had STAI scores above 39, indicating likelihood of anxiety. Ten percent of participants had CES-D scores and STAI scores indicating both likelihood of depression and anxiety and just 20% of participants scored into a racial identity development stage lower than internalization.

In the past year 95% of participants had experienced discrimination and harassment (DH) at least once, and on average 11% of participants experienced DH weekly, and 5.3% experienced DH daily. Of those experiencing any DH in the past year, 44% indicated their race as being primarily involved in the majority of DH they had experienced in the past year and 32% indicated both race and sexuality as being primarily involved in the majority of DH they had experienced (Table 2). In the past year, 52% of participants had experienced DH in public places; 43% had experienced DH in retail, customer services, or other business settings and 35% in the criminal justice system. Of those experiencing DH in public places and retail/customer service, 35% and 46%, respectively, indicated both their race and sexuality together as being primarily involved and of those experiencing DH in the criminal justice system, 62% indicated that their race was primarily involved.

### 3.2. Regression Analysis

Initially, all base variables were entered into a regression model predicting CES-D and STAI scores, with interaction terms added last, in order to determine statistical associations for the full theorized model before beta coefficient significance, strength of dependent and independent variable correlations, and amount of variance accounted for were taken into consideration in specifying the final model. Following this, stepwise independent multiple regression analysis was used to estimate the most parsimonious linear relationship between scores on the CES-D and STAI independently and internalized homonegativity, racial identity development, DH, and intersection and interaction terms. To include a proxy measure of racial and sexual identity intersectionality, an internalized homonegativity and racial identity development interaction term was included in the regression equation. The IHNI subscales for this interaction term were reverse coded, such that higher total scores on the IHNI reflect less internalized homonegativity. 

It was theorized that those possessing both higher levels of internalized homonegativity and lower levels of positive racial identity would experience greater levels of depression and anxiety than those with lower levels of internalized homonegativity and greater levels of positive racial identity. We recognize that inclusion of this interaction assumes additive identity properties and is not a true measure of intersectional identity, but in absence of any available better quantitative measure authors assessed it necessary to include this proxy term at minimum so as not to ignore the role of intersectional identity. Additionally, research suggests that coping level may moderate the relationships between IHNI, RIAS, IHNI-RIAS, and PEDQ and CES-D and STAI scores; therefore coping and coping-independent variable interaction terms were also included in the regression equation. Internalized homonegativity and Black identity achievement are both rooted in a developmental framework, and therefore age, which is closely related to maturation and identity development, was included in the equation as a covariate. 

Likewise, a social determinant of health, socioeconomic status, was included in the equation as a covariate. Education and income, which influence resource acquisition, were used to operationalize socioeconomic status. The variables age, education, income, RIAS, IHNI, RIAS-IHNI intersection, PEDQ, coping, and four coping interactions (c-RIAS, c-IHNI, c-RIAS-IHNI, c-PEDQ) were selected to test for inclusion in the model (Table 3). Cases with missing data were excluded listwise.

The variables education, income, RIAS, coping, RIAS-IHNI intersection, and c-RIAS interaction were significantly negatively correlated with CES-D scores (*P* < .05), and the variables IHNI, PEDQ, and c-PEDQ interaction were significantly positively correlated with CES-D scores (*P* < .05). The variables age, c-IHNI, and c-RIAS-IHNI were not significantly correlated with CES-D scores. The variables RIAS, coping, and c-RIAS interaction were significantly negatively correlated with STAI scores (*P* < .01) and the variables IHNI, PEDQ, RIAS-IHNI intersection, and c-PEDQ interaction were significantly positively correlated with STAI scores (*P* < .01). The variables education, age, income, c-IHNI, and c-RIAS-IHNI were not significantly correlated with STAI scores.

With the base variables education, age, income, RIAS, IHNI, PEDQ, coping, and RIAS-IHNI entered into regression models (CESD, *F* = 11.73, *P* < .01; STAI, *F* = 7.38, *P* < .01), the beta coefficients for IHNI and PEDQ were significant (*P* < .05) for both models predicting CES-D and STAI scores. Every one unit increase in the IHNI accounted for a  .63 increase in CES-D scores and a  .7 increase in STAI scores, and every one unit increase in PEDQ accounted for a  .23 increase in CES-D scores and a  .25 increase in STAI scores. After the interaction terms were added to the models (CESD, *F* = 10.79, *P* < .01; STAI, *F* = 7.06, *P* < .01), the beta coefficients for the IHNI and RIAS-IHNI intersection were significant (*P* < .05) for both models predicting CES-D and STAI scores. Every one unit increase in the IHNI corresponded to a 1.97 increase in CES-D scores and a 3.47 increase in STAI scores, and every one unit increase in the RIAS-IHNI intersection corresponded to a .71 decrease in CES-D scores and a  .94 decrease in STAI scores.

The PEDQ beta coefficient became nonsignificant, and the RIAS beta coefficient remained nonsignificant for both models predicting CES-D and STAI scores even though the RIAS-IHNI intersection beta coefficient was significant. This suggests that the IHNI variable was driving the significance of the RIAS-IHNI intersection beta coefficient. When the RIAS, IHNI, and the RIAS-IHNI intersection were entered into models, both the RIAS and the RIAS-IHNI intersection beta coefficients became nonsignificant for both models predicting CES-D and STAI scores (not shown). Only the IHNI beta coefficient remained significant.

Using stepwise multiple regression analysis the variables PEDQ and IHNI were entered into the final model for both the CES-D and STAI while age, education, income, RIAS, the RIAS-IHNI intersection, coping, and the four coping interaction terms (c-RIAS, c-RIAS-IHNI, c-IHNI, c-PEDQ) were excluded (Table 4). Taking into account the number of variables in the model and the number of observations, 64% of the variance in CES-D scores and 53% of the variance in STAI scores were explained by PEDQ and IHNI together. PEDQ alone accounted for 51% of the variance in CES-D scores, with IHNI accounting for an additional 13%. IHNI alone accounted for 46% of the variance in STAI scores, with PEDQ accounting for an additional 7%.

The overall models were significant (CES-D, *F* = 47.89, *P* < .001; STAI, *F* = 31.10, *P* < .001); there was a linear relationship between PEDQ and IHNI and CES-D and STAI scores. Holding IHNI constant, for every 1 unit increase in PEDQ, CES-D scores increased by  .29, and holding PEDQ constant, for every 1 unit increase in IHNI, CES-D scores increased by  .33. Holding PEDQ constant, for every 1 unit increase in IHNI, STAI scores increased by  .41, and holding IHNI constant, for every 1 unit increase in PEDQ, STAI scores increased by .24.

## 4. Discussion

Experience of DH and internalized homonegativity explained a large portion of the variance in depression and anxiety among this sample, as in other studies [13–15, 21, 24, 44–47]. Though experience of DH explained more of the variance in depression than internalized homonegativity and the reverse was true for anxiety, both DH and internalized homonegativity were very strongly associated with both depression and anxiety. A high percentage of the sample screened positive for likelihood of both depression (30%) and anxiety (33%), far higher percentages than in the general population (estimated between 9.3–21% for depression and 11–29% for anxiety) [1, 2] and higher than the 22% found for depression and comparable to the 36.7% found for anxiety among Black gay, lesbian, and bisexual respondents by Meyer et al. [48] and the 32.6% found for depression by Cochran and Mays [9]. The average CES-D score was 13.78 and the average STAI score was 38.30, which are roughly equal to the 13.96 and 37.5, respectively, found among a sample of similar aged BSMM in the study by David and Knight [30].

Discrimination and harassment appeared to be chronic among participants in the current study. In the past year, they reported experiencing more DH and experiencing DH more often than reported in samples of predominantly White sexual minority men (which range from 3.7% to 76%) [7, 12, 49]. Race independently and race and sexuality together were implicated most by participants as driving factors in their experience of DH. This may be further evidence of the essential role of intersectionality in understanding and contextualizing the relationship between DH and mental health outcomes among BSMM. 

Participants experienced DH most often in public places, retail settings, and the criminal justice system. This finding differs somewhat from the original PEDQ-CV validation study, in which settings with the most reported DH included public places, work, and school. The differences, however, may be a result of the inclusion of Latino and women subgroups in the validation study since their experiences may differ from those of BSMM. Most participants in this study indicated both race and sexuality together as being primarily involved in their experiences of DH in most community settings, except in the criminal justice system, where participants cited race most often, and in religious institutions, where participants cited sexuality most often. Racial identity development did not appear to play a significant role in depression and anxiety in this sample, though the lack of variability in stage placement of participants may in part explain this. 

An overwhelming majority of participants fell in the immersion and internalization stages, while very few participants were in the preencounter or encounter stages. Perhaps consequently, neither the racial identity development, identity development intersection, nor their coping interaction measures made it into the final models. Given the moderately strong univariate associations between both depression and anxiety and the race and sexual identity intersection measure, and the significant RIAS-IHNI intersection beta coefficient when all variables were entered into a model without forcing any variables to drop, possibly a different or better measure of Black identity development would have produced different results when forcing variables with nonsignificant coefficients to drop from the model. It appears that either the sample distribution was skewed on stage placement or the measure was not able to adequately differentiate participants across the stages. However, if racial identity development does not in fact play a major role in explaining depression or anxiety outcomes among BSMM, this finding does not support previous findings in the literature on racial identity development as a predictor of mental health outcomes among Black men with undocumented sexuality [20, 50]. 

Level of positive coping was not a significant indicator of depression and anxiety in this sample and thus neither confirm findings among samples of predominately White gay men [51] nor the study conducted by Peterson et al. [26]. This finding is similar to that of David and Knight [30]. In light of these findings, we share David and Knight's [30] conclusions that perhaps resiliency may be a more important mitigating factor for depression and anxiety than positive coping. Age and income were excluded variables that were close to being included in the final model for depression, and age and Black identity achievement variables were close to being included in the final model for anxiety. Perhaps with a larger sample size, and thus greater power, the relationship between depression and age and income, and anxiety and age and Black identity would be significant.

Additional limitations in this study include the use of snowball sampling, which can produce samples that may not be accurate reflections of the target population, and thus the results may not be indicative of the actual trends within the target population. Also, given the small sample size coupled with stepwise regression analysis and a high number of independent variables for possible model inclusion, power is low, *R* squared values may be overestimated, and confidence intervals for effects and predicted values may be overly narrow. Further research should focus on qualitative and quantitative exploration of the causes of VDH in community settings in which VDH is most prevalent, development and validation of tools for use among BSMM, and further examination of identity development among BSMM, including the influence of spirituality and religious institutions. 

The findings of this study suggest that efforts should be increased to implement antidiscriminatory policies in the community settings where VDH is most prevalent, public health practitioners should work to decrease negative attitudes and beliefs regarding ethnic and sexual minority identities, and service providers should help clients alleviate their internalized homonegativity and avoid VDH. Additional implications of findings for treatment and public health initiatives include training clinicians on signs and symptoms of depression and anxiety among BSMM, expansion of mental health services for this subpopulation, and social marketing campaigns that target internalized homonegativity. This inquiry sought to produce scientific evidence that could inform health and quality of life promotion related to VDH and identity development. Findings of this study further develop the conceptual framework of mental disorder acquisition by BSMM.

## Figures and Tables

**Table 1:** Demographics.

	*N*	%
Sexual orientation		
Homosexual	47	77
Bisexual	14	23
Sexual identity		
Gay	46	77
Same-gender-loving	8	13
In the life	1	2
Bisexual	3	5
Other	2	3
Highest level of education		
High school	7	12
Some college/2 yr degree	24	39
4 yr degree	19	31
Terminal degree	11	18
Religious affiliation		
Christian	30	50
Spiritual	20	33
None	10	17
Depression diagnosis	20	33
CES-D Score >15	18	30
STAI Score >39	20	33

*N* = 61. Age range = 19–50 years. Mean age = 30.7 years.

Annual income range = 0–$68,000. Mean income = $25,275.

**Table 2:** VDH, Identity, and Sector.

	*N*	%
VDH—Implicated Identity		
Race primary	25	44
Sexuality primary	11	19
Race & Sexuality primary	18	32
Cannot tell which	3	5
VDH—Sector Prevalence		
Public	31	52
Retail	26	43
Criminal justice	21	35
Entertainment Venues	20	33
Religious Institutions	19	32
Workplace/Job	19	32
School/College	18	30
Medical Services	14	23
VDH—Sector—Identity		
Public/Both	11	35
Retail/Both	12	46
Criminal justice/Race	13	62
Entertainment/Both	11	55
Religious/Sexuality	11	58
Workplace/Both	10	53
School/Both	10	56
Medical Services/Both	8	23

Proportion of participants indicating which identity component was primarily involved in the majority of VDH experienced in the previous year, community sectors with the highest prevalence of VDH, and primary identity component implicated in VDH across sector.

**Table 3:** Mean Scores and Standard Deviations of Dependent and Selected Predictor Variables.

Analysis Measure	Mean	Std. Dev.
CES-D	13.78	12.67
STAI	38.30	13.97
PEDQ	11.24	20.50
IHNI	30.98	17.21
RIAS	3.67	.82
Cope	54.46	13.32

*N* = 54.

**Table 4:** Summary of Stepwise Multiple Linear Regression Analysis.

Predictor Variable	Step	*R*	Adj. *R* ^2^	Δ*R* ^2^	Sig. Δ*R* ^2^	*β*	T	*P*
Depression								
PEDQ	1	.72	.51			.47	4.67	<.001
IHNI	2	.81	.64	.13	<.001	.45	4.44	<.001
Anxiety								
IHNI	1	.47	.46			.49	4.30	<.001
PEDQ	2	.55	.53	.07	.004	.34	3.01	.004

PEDQ: Perceived Ethnic Discrimination Questionnaire; IHNI: Internalized Homonegativity Inventory. The Beta listed is the standardized value.
